# The Glyceraldehyde-3-Phosphate Dehydrogenase and the Small GTPase Rab 2 Are Crucial for *Brucella* Replication

**DOI:** 10.1371/journal.ppat.1000487

**Published:** 2009-06-26

**Authors:** Emilie Fugier, Suzana P. Salcedo, Chantal de Chastellier, Matthieu Pophillat, Alexandre Muller, Vilma Arce-Gorvel, Patrick Fourquet, Jean-Pierre Gorvel

**Affiliations:** 1 Aix Marseille Université, Faculté des Sciences de Luminy, Centre d'Immunologie de Marseille-Luminy (CIML), UMR6546, Marseille, France; 2 Institut National de la Santé et de la Recherche Médicale (INSERM), U631, Marseille, France; 3 Centre National de la Recherche Scientifique (CNRS), UMR6102, Marseille, France; Massachusetts General Hospital, United States of America

## Abstract

The intracellular pathogen *Brucella abortus* survives and replicates inside host cells within an endoplasmic reticulum (ER)-derived replicative organelle named the “*Brucella*-containing vacuole” (BCV). Here, we developed a subcellular fractionation method to isolate BCVs and characterize for the first time the protein composition of its replicative niche. After identification of BCV membrane proteins by 2 dimensional (2D) gel electrophoresis and mass spectrometry, we focused on two eukaryotic proteins: the glyceraldehyde-3-phosphate dehydrogenase (GAPDH) and the small GTPase Rab 2 recruited to the vacuolar membrane of *Brucella*. These proteins were previously described to localize on vesicular and tubular clusters (VTC) and to regulate the VTC membrane traffic between the endoplasmic reticulum (ER) and the Golgi. Inhibition of either GAPDH or Rab 2 expression by small interfering RNA strongly inhibited *B. abortus* replication. Consistent with this result, inhibition of other partners of GAPDH and Rab 2, such as COPI and PKC ι, reduced *B. abortus* replication. Furthermore, blockage of Rab 2 GTPase in a GDP-locked form also inhibited *B. abortus* replication. Bacteria did not fuse with the ER and instead remained in lysosomal-associated membrane vacuoles. These results reveal an essential role for GAPDH and the small GTPase Rab 2 in *B. abortus* virulence within host cells.

## Author Summary

A key determinant for intracellular pathogenic bacteria to ensure their virulence within host cells is their ability to bypass the endocytic pathway and to reach a safe replication niche. *Brucella* bacteria reach the endoplasmic reticulum (ER) to create their replicating niche called the *Brucella*-containing vacuole (BCV). The ER is a suitable strategic place for pathogenic *Brucella*. Bacteria can be hidden from host cell defences to persist within the host, and can take advantage of the membrane reservoir delivered by the ER to replicate. Interactions between BCV and the ER lead to the presence of ER proteins on the BCV membrane. Currently, no other proteins (eukaryotic or prokaryotic) have yet been associated with the BCV membrane. Here we show that non-ER related proteins are also present on the BCV membrane, in particular, the glyceraldehyde-3-phosphate dehydrogenase (GAPDH) and the small GTPase Rab 2 known to be located on secretory vesicles that traffic between the ER and the Golgi apparatus. GAPDH and the small GTPase Rab 2 are involved in *Brucella* replication at late post-infection. Similarly, integrity of secretory vesicle trafficking is also necessary for *Brucella* replication. Here, we show that recruitment of the two eukaryotic proteins GAPDH and Rab 2 on BCV membranes is necessary for the establishment of the replicative niche by sustaining interactions between the ER and secretory membrane vesicles.

## Introduction

*Brucella abortus* invades both phagocytic and non-phagocytic cells [1]–[6] residing inside a membrane-bound compartment called the *Brucella*-containing vacuole (BCV). Bacteria ensure their survival and replication within host cells by avoiding fusion with lysosomes and by controlling interactions with the endoplasmic reticulum (ER) [1],[5]. The membrane of the BCV is converted into an ER-derived organelle that is permissive for replication [1]. Interactions between BCV and ER occur at dynamic membrane complexes named ERES for ER exit sites, where membrane fusion and fission events take place. These events are regulated by the small GTPase Sar 1. Sar 1 controls the assembly of COPII complexes on the ER mediating vesiculation and tubulation of the ER membrane towards the Golgi apparatus [7]–[9]. These events were shown to be essential for *B. abortus* intracellular replication at early stages of infection [10]. The *Brucella* replicative organelle has been, until now, characterized by the presence of ER chaperones such as calnexin, calreticulin, the translocator sec61β, and the ER resident enzyme protein disulfide-isomerase PDI [1],[5],[11]. Aside from these ER resident proteins, no other eukaryotic or prokaryotic proteins have been associated with the BCV membrane as yet, identification of these proteins is essential to understand how *Brucella* maintains interactions with the ER and keeps replicating within this compartment. In this work, we investigated by proteomic approaches, the composition of the BCV membrane and characterized 2 eukaryotic proteins that are essential for *B. abortus* survival. We modified a fractionation method, initially used to analyse latex bead-containing phagosomes [12],[13], to isolate BCVs obtained from cells infected with *B. abortus*. Mass spectrometry analysis of BCV proteins separated by two-dimensional (2D) gel electrophoresis revealed the presence of the eukaryotic protein GAPDH (glyceraldehyde-3-phosphate dehydrogenase). Further work on GAPDH revealed a role for GAPDH and the small GTPase Rab 2 in the intracellular replication of *B. abortus*.

## Results

### Survival and Replication of *B. abortus* inside BHK-21 Cells

**Figure 1:**
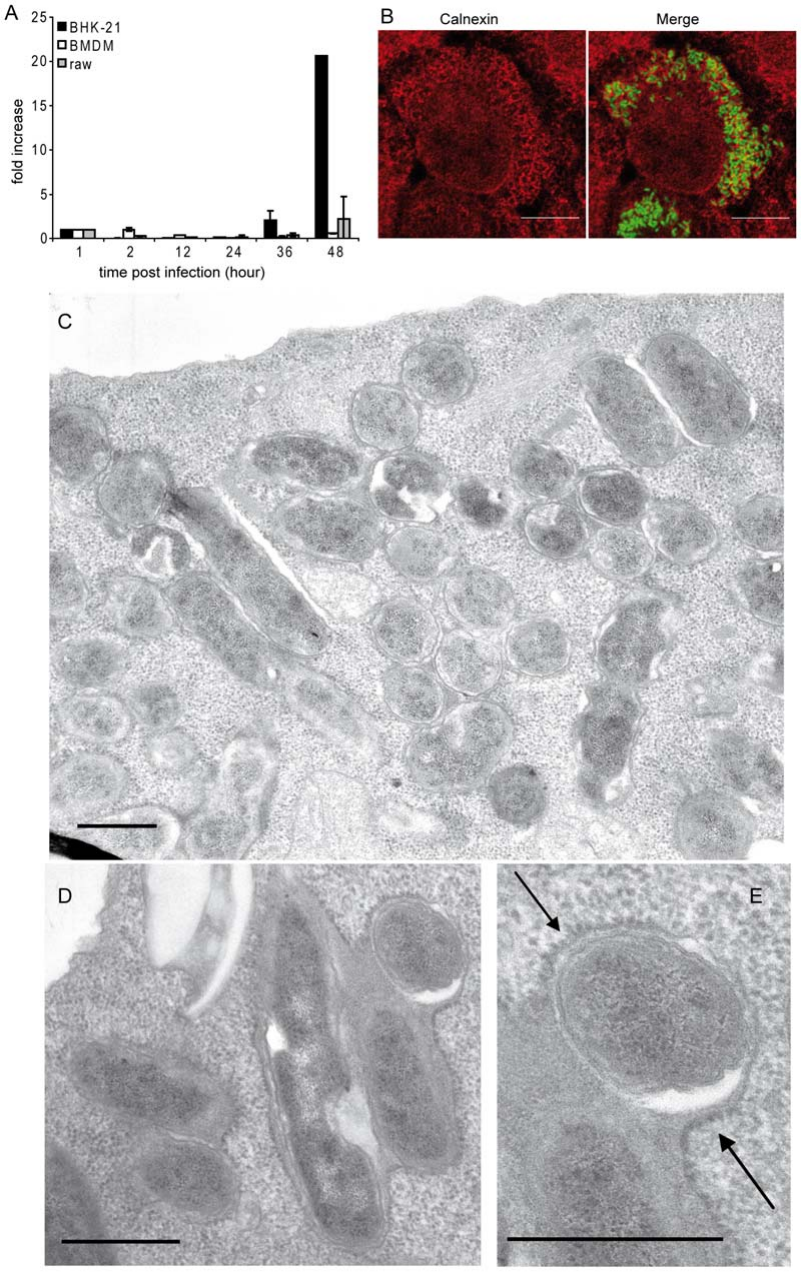
Survival and replication of *B. abortus* inside BHK-21 cells. (A) Fold increase of intracellular replication of *B. abortus* over 48 h within 3 different cell types: BHK-21 fibroblasts, BMDM and Raw 264.7 macrophages. Data are means±SD of three independent experiments. (B) Confocal microscopy micrographs of BHK-21 infected for 48 h with *B. abortus* expressing GFP. Fixed cells were stained with anti-calnexin antibody (red) to label the ER. (Scale bars: 10 µm). (C) Electron microscopy micrographs of thin sections of BHK-21 cells infected with *B. abortus* and fixed 48 h later. General view of an infected cell displaying several BCVs. Note that most BCVs contain more than one bacterium. (D) Enlarged view of a BCV containing several bacteria. (E) Enlarged view of part of the BCV shown in *D*. Note the ribosomes along the BCV membrane (arrows). (Scale bars: 0.5 µm).

To analyse the protein composition of the BCV membrane, a large number of purified BCVs is required. We first tried to determine which cell type was more susceptible for *B. abortus* infection by monitoring the infection of primary phagocytic cells (bone marrow-derived macrophages: BMDM) and phagocytic and non phagocytic cell lines (Raw 264.7, HeLa, baby hamster kidney: BHK-21). Although no difference was observed in the percentage of infected cells at 48 h post-infection (p.i.) between the different cell types (nearly 35% infected cells), the intracellular replication of *B. abortus* was 10 times higher within BHK-21 cells than in the other cell types (Figure 1A).

As in macrophages [1] and in dendritic cells [14], *B. abortus* GFP (in green) in BHK-21 cells were located inside a membrane-bound vacuole labelled with the ER marker calnexin (in red), suggesting that *B. abortus* replicates in ER-derived compartments within BHK-21 (Figure 1B). This result was confirmed by electron microscopy analysis of infected cells (Figures 1C, 1D and 1E). *B. abortus* was located inside a membrane-bound compartment resembling the ER with ribosomes lining the vacuolar membrane. However, unlike what has previously been described in BMDM and HeLa cells [1],[5], several bacteria resided inside a unique vacuole (Figures 1C and 1D). This may explain the increase of *B. abortus* intracellular replication within BHK-21 cells. Taken together, these results show that *B. abortus* extensively replicates in an ER-derived compartment in BHK-21 cells, validating BHK-21 cells as a good cell model for studying the proteic composition of BCV membranes.

### Isolation of BCVs

**Figure 2:**
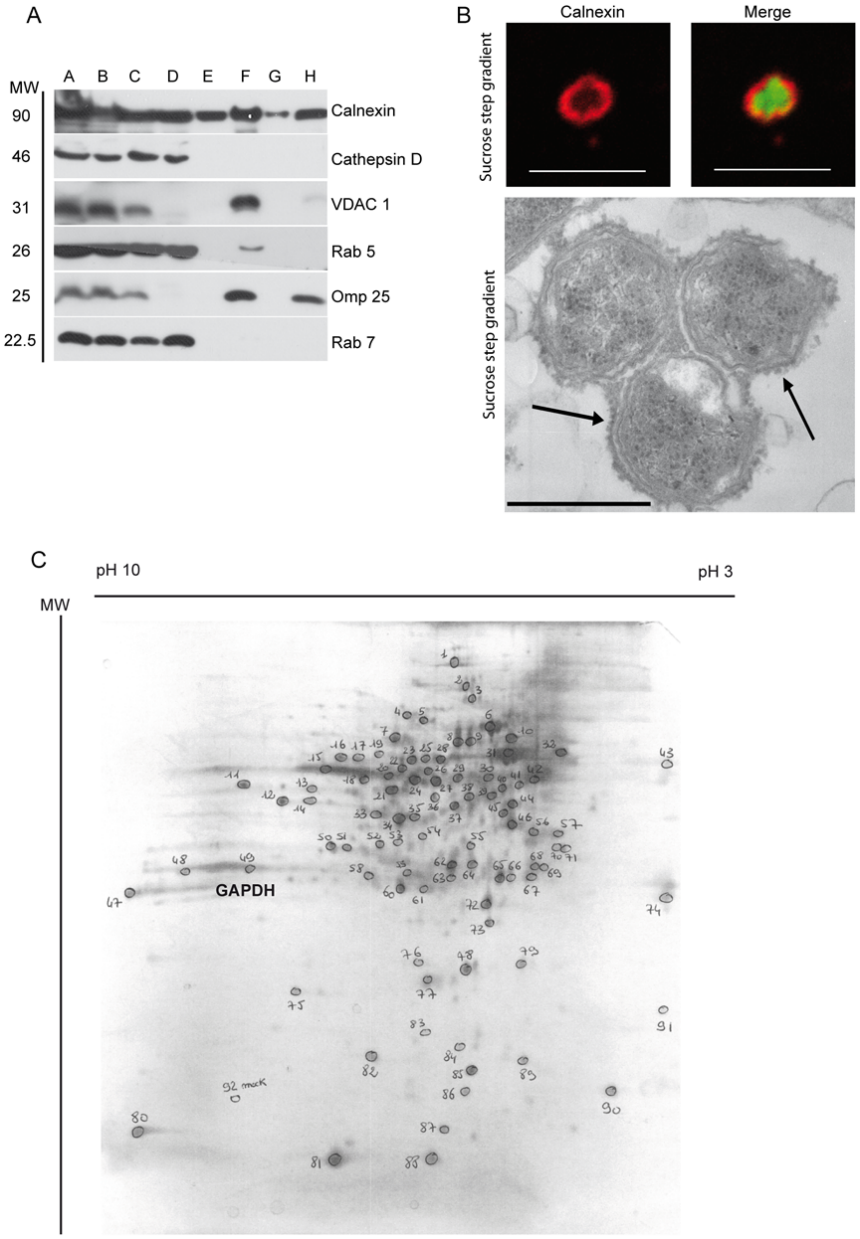
Biochemical and microscopic analysis of purified BCV. (A) Western blotting of each step of the fractionation method: A/Intact BHK-21, B/BHK-21 homogenate, C/PNS, D/Supernatant after sucrose gradient, E/Sucrose 12%, F/Interface 50%–12%, G/Sucrose 50%, H/Sucrose step gradient. Immunoblotting with anti-calnexin, -cathepsin D, -VDAC 1, -Rab 5, -Omp 25 and -Rab 7 antibodies. (B) Confocal microscopy micrographs of isolated BCVs from the PNS and the sucrose step gradient. *B. abortus* express GFP (green) and the ER is labelled with anti-calnexin antibody (red). (Scale bars: 5 µm). Electron microscopy micrographs of thin sections of isolated BCVs within the sucrose step gradient. The ribosomes along the BCV membrane are indicated by arrows. They seem to be as abundant along the BCV membrane as in intact cells (Scale bars: 0.5 µm). (C) 2D gel electrophoresis of BCVs membrane stained with PlusOne Silver Staining Kit. Each spot analysed by Mass Spectrometry is encircled and annotated with the corresponding number.

In order to obtain high concentration of membrane proteins, we optimized a fractionation method to isolate and purify BCVs. Within the post-nuclear supernatant (PNS) of BHK-21 cells infected with *B. abortus* for 48 h, approximately 1.5% of vesicles were GFP positive as detected by flow cytometry and we found that 67% of BCVs remained positive for the ER marker calnexin (Figure S1). After PNS preparation, BCVs were first purified on a 50%–12% sucrose gradient. BCVs were present at the interface, which corresponded to 37% sucrose as indicated by the densitometer measurement (data not shown). Bacteria were only detected in the interface fraction (Figure 2A, lane F) by the presence of the *Brucella* transmembrane outer membrane protein Omp 25 [15]. On the contrary, ER was detected in each fraction of the sucrose gradient with an anti-calnexin antibody (Figure 2A).

This first purification step allowed the elimination of Rab 7-positive late endosomes and cathepsin D-positive lysosomes from the BCV membrane fraction (Figure 2A, lane F). The interface fraction was then loaded onto a second sucrose step gradient. This gradient allowed the removal of early endosomes (Figure 2A, lane H) and ER structures non-associated with the BCV as detected by immunofluorescence and electron microscopy (data not shown). Although the fraction of BCVs was now devoid of endocytic organelles (Figure 2A, lane H), a few mitochondria detected by the anti-mitochondrial VDAC 1 protein antibody were still present. These could be eliminated by incubating the interface fraction with dynabeads (M-500 subcellular) coated with anti-VDAC 1 antibody, but most of BCVs were lost (data not shown). As a consequence, for proteomic analysis we chose not add the dynabeads step. We analysed BCVs by immunofluorescence and electron microscopy to determine if BCVs were still intact after fractionation. 77% of *B. abortus* GFP were surrounded by ER-positive vacuoles (Figure 2B). Electron microscopy analysis showed that BCVs were still intact after subcellular fractionation (Figure 2B). Bacteria were surrounded by an ER membrane-bound compartment and residual ribosomes were still located on the ER vacuolar membrane. Taken together, these data indicate that we successfully isolated BCVs and preserved their membrane integrity.

### Determination of BCV protein composition

To determine the protein composition of the BCV membrane, vacuolar proteins were solubilized by a mild Triton X-100 treatment, precipitated by a trichloroacetic acid/acetone and analysed by 2D gel electrophoresis. Approximately one hundred spots were detected by silver gel staining (Figure 2C) and further analysed by mass spectrometry. Proteins of the BCV membrane identified by mass spectrometry are listed in Table S1. Spot numbers on 2D gel (Figure 2C) correspond to the numbers of identified proteins listed in Table S1. As expected, 18% of proteins identified were ER proteins (i.e. calreticulin, ERP 57 and PDI) and 13% ribosomal proteins confirming that the replicative niche of *B. abortus* is derived from the ER. Interestingly, 29% of proteins were not ER-related proteins (19% bacterial and 10% eukaryotic proteins). The remaining 40% proteins were mitochondrial proteins. Among the identified proteins, we focused on one peculiar eukaryotic protein: the Glyceraldehyde-3-phosphate dehydrogenase (GAPDH) annotated by spot number 49 on the 2D gel (Figure 2C) and in Table S1. GAPDH has multiple functions within host cells such as glycolysis and apoptosis [16],[17] More interestingly, GAPDH interacts with the small GTPase Rab 2 to control vesicular retrograde transport between the ER and the Golgi [18]. The presence of GAPDH on the BCV membrane leads us to hypothesise that vesicular retrograde transport may be involved in *Brucella* replication within the ER. Therefore, we investigated the role of GAPDH and Rab 2 in *Brucella* survival within host cells.

### GAPDH and Rab 2 are present on BCV membranes

**Figure 3:**
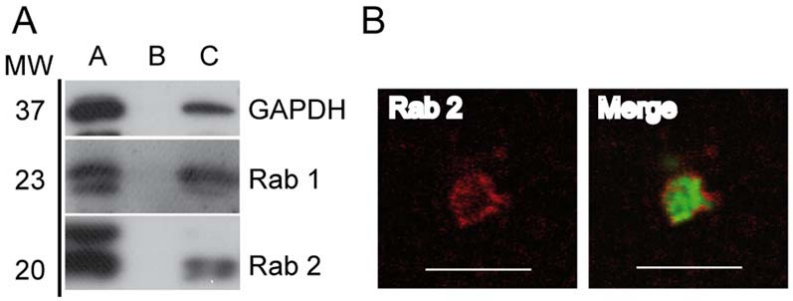
GAPDH and Rab 2 localise to the BCV membrane. (A) Western blotting of the enriched fraction of BCVs: A/Interface 50%–12%, B/Sucrose 50%, C/Sucrose step gradient. Immunoblotting with anti-GAPDH, anti-Rab 1 and anti-Rab 2 antibodies. (B) Confocal microscopy micrographs of isolated BCVs from the sucrose step gradient. *B. abortus* GFP (green) and Rab 2 staining (red). (Scale bars: 5 µm).

First, the presence of GAPDH on the BCV membrane was confirmed by immunoblotting (Figure 3A, lane C). Its partner, the small GTPase Rab 2, was also detected in the enriched BCV fraction (Figure 3A, lane C). The small GTPase Rab 1, which is known to be a resident of the ER as well as vesicular and tubular clusters (VTCs) and Golgi apparatus [19],[20] was also detected in BCVs (Figure 3A, lane C). As no commercial antibody was suitable for immunofluorescence detection of GAPDH on BCVs obtained from BHK-21 cells, we analysed the presence of its partner the small GTPase Rab 2. We found that 35% of isolated BCVs were surrounded by Rab 2 staining (Figure 3B). Together these results confirm the presence of GAPDH and Rab 2 on BCVs. We further studied the role of Rab 2 and GAPDH in HeLa cells, a well-established cell culture model for *Brucella* infection.

### Rab 2 is involved in intracellular replication of *B. abortus*

**Figure 4:**
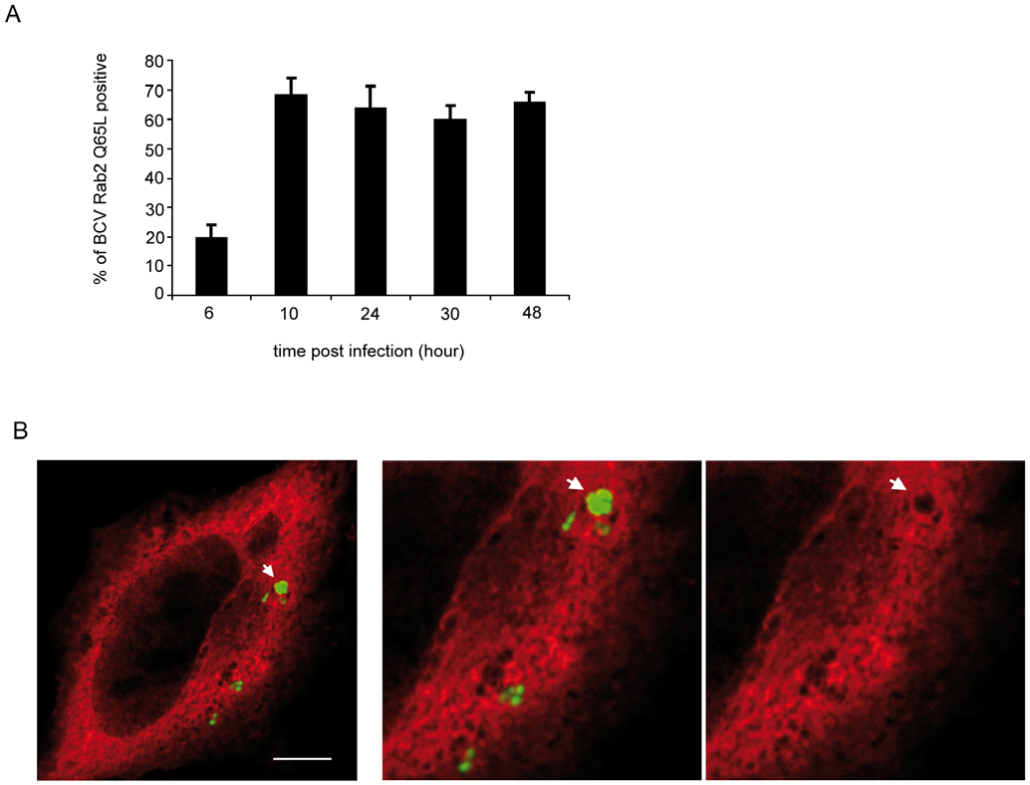
Recruitment of the active form of Rab 2 (Rab 2 Q65L) on BCV. (A) Quantification of the percentage of BCV positive in myc Rab 2 Q65L transfected HeLa cells and infected with *B. abortus* GFP at different time p.i. (B) Confocal micrographs of a myc Rab 2 Q65L transfected Hela cell infected with *B. abortus* GFP at 10 h p.i. The myc tag was immunostained with anti-myc antibody. The arrow indicates myc Rab 2 Q65L accumulation around BCVs. (Scale bar: 10 µm).

We showed that the GTPase Rab 2 is present on the vacuolar membrane of purified BCVs at 48 h p.i. (Figure 3A and 3B). In order to determine the kinetics of Rab 2 acquisition on BCV membranes, we first examined the presence of endogenous Rab 2 on BCVs within infected HeLa cells. Although we could detect Rab 2 on isolated BCVs, labelling of infected cells was extremely weak. As a consequence, we overexpressed Rab 2 within HeLa cells by using a dominant positive form of Rab 2: Rab 2 Q65L, which corresponds to Rab 2 locked in its GTP-bound form. HeLa cells were transfected with Myc Rab 2 dominant positive Q65L for 24 h and then infected with *B. abortus* GFP. The intracellular replication of *Brucella* within cells transfected or not with Q65L Rab 2 was similar (data not shown). Figure 4A represents the quantification of BCVs positive for Rab 2 Q65L at different times p.i. At 6 h p.i. few BCVs were surrounded by the active form of Rab 2 (20±4.2%). Then, at 10 h p.i. 68.4±5.7% of BCV had acquired Rab 2 Q65L and remained positive for Rab 2 Q65L until 48 h p.i. Figure 4B illustrates the recruitment of Rab 2 on BCVs at 10 h p.i. as indicated by the arrow. These results indicate that the recruitment of the active form of Rab 2 takes place just before the interaction of BCVs with the ER, known to occur around 12 h p.i [5]. Indeed, most of BCVs surrounded by Rab 2 Q65L were still LAMP-1-positive at 10 h p.i. (data not shown).

**Figure 5:**
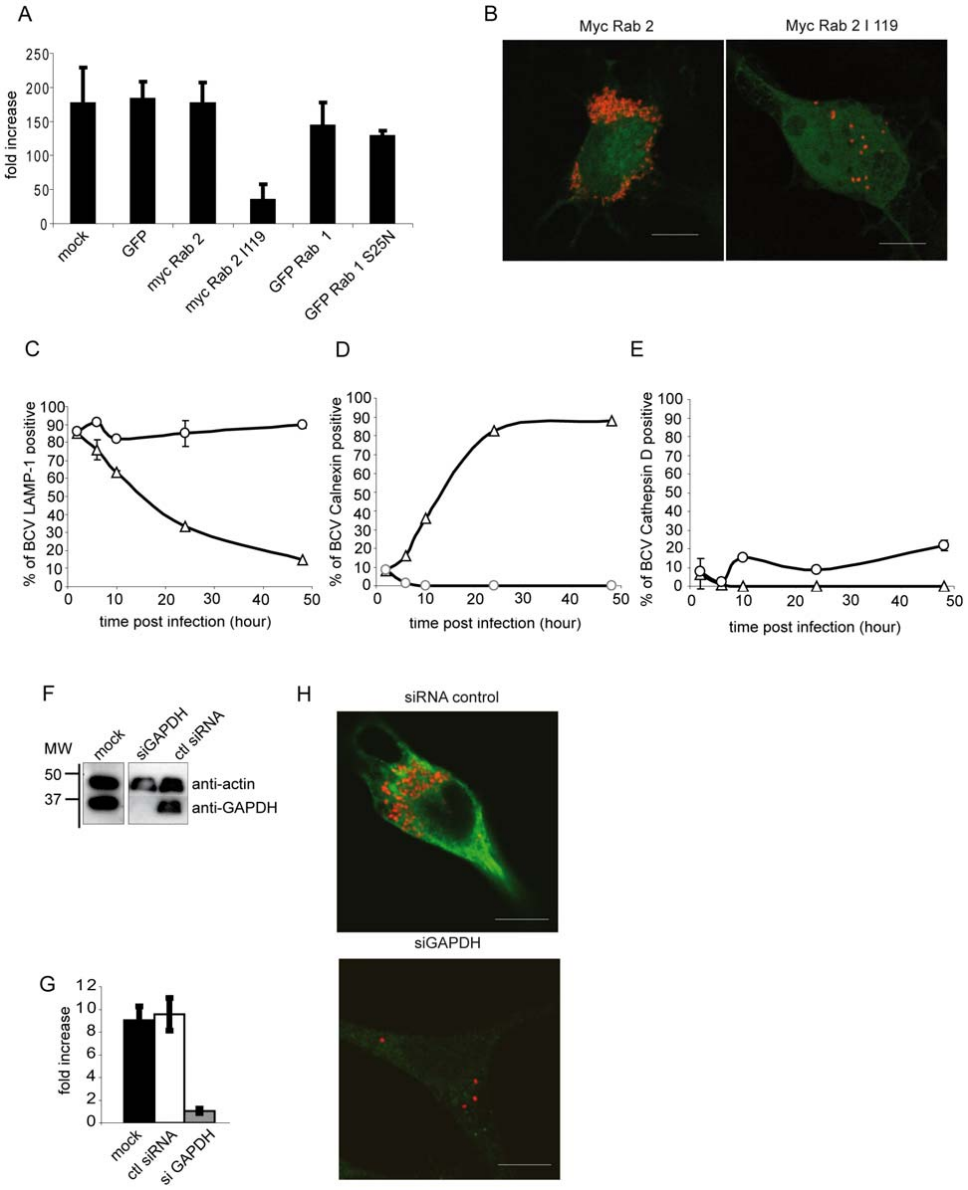
The inhibition of GAPDH expression and the dominant negative of Rab 2 affects *B. abortus* replication at 48 h p.i. (A) Fold increase of intracellular CFUs at 48 h p.i. of Hela cells infected with *B. abortus* Ds Red. Infected cells were transfected or not with GFP, myc Rab 2, myc Rab 2 I119, GFP Rab 1 or GFP Rab 1 S25N at 2 h p.i. Data are means±SD of three independent experiments. (B) Confocal micrographs of Hela cells infected with *B. abortus* Ds red and transfected with myc Rab 2 or its dominant negative myc Rab 2 I119. The myc tag was immunostained with anti-myc antibody (Scale bars: 10 µm). (C, D, E) Quantification of the percentage of BCV positive for LAMP-1 (C), Calnexin (D) or Cathepsin D (E) in myc Rab 2 I119 transfected HeLa cells (open circle) or not (open square) and infected with *B. abortus* wild-type. (F) Western blotting of cellular extracts from cells transfected with GAPDH siRNA or siRNA control. Immunoblotting with anti-GAPDH and anti-actin antibodies. (G) Fold increase of intracellular CFUs at 48 h p.i. of transfected Hela cells with GAPDH siRNA or siRNA control and infected with *B. abortus*. Data are means±SD of three independent experiments. (H) Confocal micrographs of Hela cells infected with *B. abortus* Ds red and transfected with GAPDH siRNA or siRNA control. GAPDH was immunostained with anti-GAPDH antibody (Scale bars: 10 µm).

Retrograde vesicle formation from the VTC is mediated by the exchange of GDP to GTP on the GTPase Rab 2 [21]. In order to inhibit the retrograde transport between the ER and the Golgi, we used a dominant negative form of Rab 2: Rab 2 I119, which corresponds to Rab 2 locked in its GDP-bound form. HeLa cells infected with *B. abortus* Ds Red were transfected with either GFP plasmid as a transfection control, myc Rab 2, myc Rab 2 dominant negative I119, GFP Rab 1 or GFP Rab 1 dominant negative S25N. Figures 5A, 5B and S2A illustrate the level of intracellular replication at 48 h p.i. in the different transfected cells. We observed extensive *Brucella* replication in control cells or cells transfected either with GFP or myc Rab 2 (Figure 5A, 5B and S2A). Interestingly, transfections with either the GFP Rab 1 or its dominant negative did not affect *Brucella* replication, contrasting with results obtained from cells infected with *Legionella pneumophila* and transfected with the dominant negative Rab 1 S25N (Figure S2) [22]. On the contrary, in cells transfected with myc Rab 2 dominant negative I119 (Figure 5A and 5B), *Brucella* replication was strongly decreased (six times) and 89.8±1.62% of the bacteria were located in a lysosomal LAMP-1-positive compartment at 48 h p.i (Figure 5C and S2B), a time point were most of the *Brucella* (88%) are within an ER-positive, LAMP-1-negative compartments (Figure 5C and 5D). This shows that in cells transfected with the Rab 2 dominant negative form, *Brucella* was not able to reach the ER. Indeed, only a small percentage of BCVs were positive for cathepsin D at 48 h p.i. (22% of BCVs within cells overexpressing Rab 2 dominant negative) (Figure 5E). In contrast 92% of heat-killed *Brucella* BCVs were already cathepsin D-positive at 2 h p.i. (data not shown). Therefore, inhibition of retrograde vesicle formation from the VTCs mediated by Rab 2 affects *Brucella* replication. Taken together these results indicate that the trafficking between ER and Golgi controlled by Rab 2 is important for entry of *Brucella* in the ER and subsequent intracellular replication.

### The early secretory pathway between ER and Golgi is involved in *Brucella* replication

To investigate the role of GAPDH on *Brucella* pathogenesis, we down-regulated the expression of GAPDH in HeLa cells infected with *B. abortus* by using small interfering RNA. HeLa cells transfected with GAPDH siRNA for 72 h efficiently and specifically reduced the expression level of GAPDH (Figures 5F and 5H), whereas, siRNA control did not affect the GAPDH expression (Figures 5F and 5H). Inhibition of GAPDH expression induced a 10 fold reduction in *Brucella* replication as compared to non-transfected cells or cells transfected with the siRNA control (Figure 5G and 5H). This result indicates that the presence of GAPDH on the BCV membrane is required for *Brucella* replication. We showed above that in cells transfected with the Rab 2 dominant negative form, *Brucella* was not able to reach the ER and remained in a LAMP-1 compartment. Similarly, in siRNA GAPDH-treated cells, *Brucella* was found in a LAMP-1-positive compartment (Figure S3). In addition, inhibition of GAPDH expression prevented Rab2 recruitment on BCVs, as shown after BCV purification (Figure S3). Quantification showed that 77% of BCVs were positive for Rab 2 in control cells whereas only 12% of siRNA GAPDH-treated cells were able to recruit Rab 2. These results show that GAPDH is an important host factor for BCV biogenesis.

**Figure 6:**
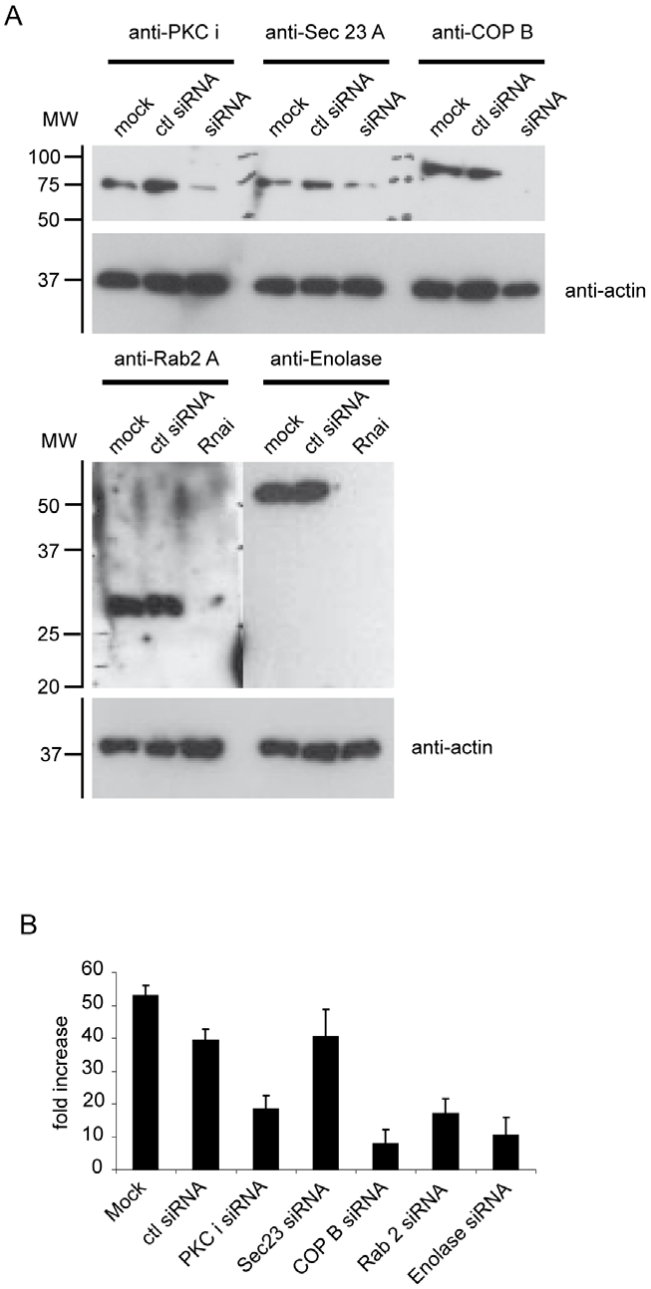
The inhibition of PKC ι/λ, COPI, Rab 2 and Enolase expression affects *B. abortus* replication at 48 h p.i. (A) Western blotting of cellular extracts from cells transfected with PKC ι siRNA, COP B siRNA, Rab 2 A siRNA, Enolase siRNA or control siRNA-A. Immunoblotting with anti-PKC ι, -COP B, -Rab 2 A, -Enolase and anti-actin antibodies. (B) Fold increase of intracellular CFUs at 48 h p.i. of transfected Hela cells with PKC ι siRNA, COP B siRNA, Rab 2 A siRNA, Enolase siRNA or control siRNA-A and infected with *B. abortus*. Data are means±SD of three independent experiments.

GAPDH is known to play several functions within host cells [16]–[18],[23]. To confirm the involvement of the retrograde transport of the early secretory pathway in *Brucella* pathogenicity, we investigated the role of other key components known to control the vesicular trafficking between ER and Golgi, such as the kinase PKC ι/λ and the coat COPI complex. Inhibition of these components was performed by infecting HeLa cells transfected with small interfering RNAs. We used a PKC ι siRNA to silence the expression of the kinase PKC ι/λ, a COP B siRNA to silence the subunit β of the COPI complex, a Rab 2 A siRNA to silence the GTPase Rab 2 and a α-Enolase siRNA to silence the Enolase, an enzyme involved in glycolysis. Cellular extracts prepared from HeLa cells transfected with the appropriate siRNA for 72 h efficiently and specifically reduced the expression of PKC ι, COPI, Rab 2 and Enolase (Figure 6A), whereas, siRNA control did not (Figure 6A). Figure 6B shows the intracellular replication of *B. abortus* at 48 h p.i. within infected-HeLa cells transfected with different siRNAs. *Brucella* replication was reduced 2.2, 2.3 and 5 fold in cells transfected with PKC ι siRNA, Rab 2 A siRNA and COP B siRNA, respectively as compared to cells transfected with the siRNA-A control. This result indicates that each member of the complex GAPDH/COPI/Rab2/PKCι/λ is required for *Brucella* replication. Surprisingly, we noticed that the intracellular replication of *Brucella* was reduced 4 fold under the inhibition of Enolase expression. This result suggests that host glycolysis is necessary for *Brucella* survival within host cells. Taken together, these results demonstrate the role played by the early secretory pathway, in particular the GAPDH/COPI/Rab2/PKCι/λ retrograde vesicles, to ensure replication within host cells at late stages of infection.

## Discussion

Many intracellular bacteria, with the aim of generating a suitable niche of replication, have been shown to alter the phagosomal membrane composition to avoid fusogenic interactions with lysosomes [24]. For example, *Salmonella typhimurium* secretes multiple effector molecules onto the vacuolar membrane (via its type III secretion systems), which interact with host proteins to modulate vesicle transport and vacuolar membrane dynamics [25]. This enables *Samonella* to replicate in a vacuole that interacts with late endosomes whilst avoiding fusion with lysosomes. In contrast, *Legionella* and *Brucella* replicate in ER-derived compartments [1],[26]. In the case of *Legionella*, several recent studies have highlighted the role of type IV secreted proteins in recruiting eukaryotic proteins to the vacuolar membrane and by mechanisms that are still unclear sustain intracellular replication [27]. Much less in known for *Brucella*, particularly regarding the membrane composition of BCVs. Previous work has demonstrated that the small GTPase Sar1 is implicated in *Brucella* intracellular survival [10]. However, apart from ER-resident proteins no other eukaryotic molecules have been associated with the BCV membrane.

Phagosomal proteomic studies using latex bead-containing phagosomes have significantly helped to decipher phagosome biology [28]. Using a modified procedure for phagosome purification, we analysed in detail the protein composition of the BCV membrane in order to identify eukaryotic proteins recruited to BCVs during intracellular replication. We developed a fractionation method to isolate intact BCVs from infected BHK-21 cells. This method allowed us to establish for the first time the BCV membrane protein map of the replicative niche of *Brucella*. As expected, a proportion of proteins on the BCV membrane were ER and ribosomal proteins.

Interestingly, one of the proteins identified was GAPDH, a non-ER eukaryotic protein which is normally located on VTCs between ER and the Golgi apparatus. This protein has been extensively studied by Tisdale et al [29]–[35]. GAPDH forms an active complex with the small GTPase Rab 2 and the protein kinase C (PKCι/λ), which is necessary for secretory vesicular transport. First studies showed that an inactive form of Rab 2 had a negative effect on anterograde transport of vesicles from the ER to the Golgi [21]. Recently, the group of Tisdale has shown that Rab 2 modulates protein retrograde transport from the Golgi to the ER by recruiting GAPDH to VTCs which allows the release of retrograde-directed vesicles [31],[35]. This retrograde transport requires a functional GAPDH/COPI/Rab2/PKCι/λ complex. Presence of GAPDH and Rab 2 on the BCV membrane suggests that *Brucella* is somehow interacting with VTCs or intercepting vesicle trafficking of the retrograde transport. Consistent with this hypothesis we found that inhibition of GAPDH resulted in reduced intracellular replication of *Brucella*. However, we cannot exclude that its role in the host cell glycolysis also contributes to the intracellular survival of *Brucella*. Indeed, silencing of enolase, another enzyme involved in glycolysis, also resulted in inhibition of *Brucella* replication. Further work is necessary to determine if *Brucella* is directly using the host glycolysis to its advantage, for example as a source of energy.

Nevertheless, the implication of retrograde transport in *Brucella* virulence is clearly demonstrated by the inhibition of the bacterial replication upon silencing of each member of the complex GAPDH/COPI/Rab2/PKCι/λ.

In addition, we found that Rab 2 is recruited on the BCV membrane before fusion with the ER suggesting that the retrograde transport might have an important role in the establishment of the bacterial replicative niche. Previous work has demonstrated that BCV-ER fusion events occur specifically at ER exit sites and are mediated by the small GTPase Sar1. This work also demonstrated that the anterograde pathway mediated by COPI/ARFI-coated vesicles are not involved in the BCV-ER fusion and that COPII complex formed on ER membranes was found in close apposition to BCV [10]. Our results also implicate the small GTPase Rab 2, in the early events of BCV biogenesis since inactivation of Rab 2 using the dominant negative prior to infection prevented fusion of BCVs with the ER. When inactivation is performed after infection, *Brucella* is able to start to replicate at 24 h (data not shown) whereas at 48 h p.i. there is a strong replication effect. Overall, these results suggest that Rab 2 is also necessary for *Brucella* survival after it has established its ER-derived replication niche.

Consistent with this hypothesis, we found that *Brucella* replication was also affected with prolonged brefeldin A treatment, which causes the Golgi apparatus to redistribute to the ER (data not shown). Regeneration of the Golgi apparatus after brefeldin A treatment allowed *Brucella* to recover and replicate (data not shown). Therefore, it is possible that trafficking of secretory vesicles from the Golgi apparatus to the ER is beneficial for *Brucella* replication. Perhaps, extensive bacterial replication requires an additional membrane input that may come from vesicles from the secretory pathway.

*Brucella* seems to manipulate host cells differently than *Legionella pneumophila*, another bacterial pathogen which replicates in the ER. Indeed, *Legionella* was shown to manipulate host cells by secreting specific type IV secretion system bacterial effectors, for example DrrA (or LidA) [36],[37]. DrrA is able to mimic the guanosine exchange factor (GEF) of the small GTPase Rab 1 to catalyse the exchange of GDP to GTP which lead to the activation and the recruitment of Rab 1 on the *Legionella*-containing vacuole [36],[37]. Interestingly, another secreted effector LepB acts like a GTPase-activating protein (GAP) which inactivates Rab 1 [38]. Inhibition of Rab1 impairs *Legionella* replication [22]. In this study, we demonstrate that Rab 1 is not involved in intracellular replication of *Brucella*.

Rab 1 is implicated in the anterograde pathway whereas Rab 2 is also essential for the retrograde pathway. After 24 h of replication within its ER-derived vacuole, *Legionella* lyses its vacuole to reach the cytosol and to infect the neighbouring host cells [39]. This suggests that *Legionella* does not need an influx of membrane since the bacteria lyse the host cell relatively quickly compared to *Brucella*, which continues to replicate within vacuoles for a long period of time (at least another 48 h) [1]. Therefore it is possible that *Brucella* might require an extensive influx of membrane for its own survival. Targeting of the retrograde pathway could ensure continued fusion with incoming vesicles and an influx of membrane.

At this stage, it is unclear how *Brucella* is controlling biogenesis of ER-derived BCVs. It is possible that some secreted effector proteins not yet identified are directly recruiting GAPDH and Rab 2 to the BCV membrane. The type IV secretion system VirB, essential for sustained interaction and fusion with ER membranes [1], could secrete these effectors to recruit Rab 2 onto the BCV membrane. We are currently undertaking further studies to identify the bacterial effector(s) which target(s) Rab 2 and GAPDH.

## Materials and Methods

### Bacterial strains

The bacterial strains used in this study were the smooth virulent *B. abortus* strain 2308 GFP [1] or 2308 Ds Red kindly provided by Jean-Jacques Letesson (URBM, Immunology Laboratory, FUNDP, Namur, Belgium). Bacteria were inoculated in tryptic soy broth (TSB. Sigma-Aldrich) with kanamycin and grown at 37°C overnight (16 h) as previously described for infections [1].

### Plasmids

GFP vector used was pEGFP-C1 (Clontech). Plasmids pGEM Rab 2, pGEM Rab 2 I119 and pGEM Rab 2 Q65L were kindly given by Craig Roy (Section of Microbial Pathogenesis, Yale University School of Medicine, New Haven, USA). The rab2 sequence from the vectors pGEM Rab 2, pGEM Rab 2 I119 and pGEM Rab 2 Q65L was amplified by PCR with two specific primers: O-403 (5′GGGGACAAGTTTGTACAAAAAAGCAGGCTTCGCGTACGCCTATCTCTTCAAGTACAT3′) and O-404 (5′GGGGACCACTTTGTACAAGAAAGCTGGGTCCTAACAGCAGCCGCCCCCAGCCTGCTG3′). The myc tag (pCMV-myc) was added by Gateway procedure according to the manufacturer's instructions (Invitrogen) to obtain myc Rab 2, myc Rab 2 I119 and myc Rab 2 Q65L. Plasmids GFP Rab 1 and GFP Rab 1 S25N were also kindly given by Craig Roy. The silencing of endogenous GAPDH was performed by small interfering RNA with the commercial siRNA GAPDH (Applied Biosystems Ambion). A negative control siRNA was also used (Applied Biosystems Ambion) as a transfection control. Silencing of endogenous PKC ι, COP B, Rab 2 A and α-Enolase were performed by small interfering RNA with the corresponding commercial siRNA (Santa Cruz Biotechnology). A negative control siRNA-A was also used (Santa Cruz Biotechnology) as a transfection control.

### Antibodies

The primary antibodies used were: a mouse monoclonal anti-actin given by Lena Alexopoulou (CIML, Marseille, France); a rabbit polyclonal anti-calnexin (Stressgen); a rabbit polyclonal anti-cathepsin D [40]; a goat polyclonal T-14 COP B (Santa Cruz Biotechnology); a mouse monoclonal anti-GAPDH (Sigma Aldrich); a rabbit polyclonal anti-giantin (Covance); a mouse monoclonal anti-GM130 (Transduction Laboratories); a rabbit polyclonal anti-LAMP-1 (Abcam); a mouse monoclonal 4A1 anti-LAMP-1 kindly given by Jean Gruenberg (University of Geneva, Switzerland); a mouse monoclonal 9E10 anti-c-myc tag (Santa Cruz Biotechnology); a mouse monoclonal anti-Omp 25 (A59/05F01/C09) kindly given by Michel Zygmund (INRA, Tours, France); a mouse monoclonal H-12 PKC ι (Santa Cruz Biotechnology); a rabbit polyclonal anti-Rab 1 (Santa Cruz Biotechnology); a polyclonal rabbit anti-Rab 2 and a mouse monoclonal anti-Rab 5 kindly given by Marino Zerial (Max Planck Institute of Molecular Cell Biology and Genetics, Dresden, Germany); a rabbit polyclonal anti-Rab 7 [40] and a rabbit polyclonal anti-VDAC 1 (Abcam). The secondary antibodies used were: goat anti-mouse HRP (Sigma Aldrich); goat anti-rabbit HRP (Sigma Aldrich) for western blotting; and donkey anti-rabbit Texas red (Jackson ImmunoResearch); donkey anti-mouse Texas red (Jackson ImmunoResearch); donkey anti-mouse Cy3 (Jackson ImmunoResearch); donkey anti-mouse FITC (Jackson ImmunoResearch); donkey anti-mouse Cy5 (Jackson ImmunoResearch); phalloidin-tetramethylrhodamine isothiocyanate (TRITC. Sigma Aldrich) for immunofluorescence and a IgG anti-rabbit coupled to phycoerytrin (PE. Serotec) for flow cytometry.

### Cell culture, infection and transfection

Baby hamster kidney (BHK-21) cells were cultured at 37°C with 5% CO_2_ atmosphere in GMEM (Glasgow's modified Eagle's medium. Gibco) supplemented with 10% Tryptose Phosphate Broth (Sigma Aldrich), 5% FCS (Perbio) and 1% L-glutamine (Gibco) and seeded 24 h before infection on 55 cm^2^ culture dishes (1.6×10^6^ cells per dish) for fractionation or at a surface ratio of 1/10 in 24-well plates containing 12-mm glass coverslips for immunofluorescence and CFUs. HeLa cells and Raw 264.7 macrophages were cultured at 37°C in a 5% CO_2_ atmosphere in DMEM supplemented with 10% FCS, 1% non-essential amino acids and 1% L-glutamine and seeded 24 h before infection at a surface ratio of 1/10 in 24-well plates containing 12-mm glass coverslips. Bone marrow-derived macrophages (BMDM) were isolated from femurs of 6 to 10-week-old C57Bl/6 female mice and differentiated into macrophages as previously described [41].

Infections were performed at a multiplicity of infection of 200∶1 by centrifuging bacteria onto BHK-21, HeLa cells, BMDM or Raw 264.7 macrophages at 400 g for 10 min at 4°C, and then by incubating the cells for 1 h (for BHK-21 and HeLa cells) or 15 min (for BMDM and Raw 264.7 macrophages) at 37°C under a 5% CO_2_ atmosphere. Cells were extensively washed with their respective medium to remove extracellular bacteria and were incubated for an additional hour in their respective medium supplemented with 50 µg/ml gentamycin to kill extracellular bacteria. Thereafter, the antibiotic concentration was decreased to 10 µg/ml.

To monitor *Brucella* intracellular survival, infected cells were washed three times with PBS and lysed with 0.1% (vol/vol) Triton X-100 in PBS. Serial dilutions in PBS of lysates were plated onto TSB agar plates to enumerate CFUs.

The expression of GFP, myc Rab 2, myc Rab 2 I119, myc Rab 2 Q65L, GFP Rab 1, GFP Rab 1 S25N were performed by transfecting HeLa cells using the FuGENE transfection reagent (Roche), according to the manufacturer's instructions. Depending of experiment, transfections were performed either 24 h before or 2 h after *Brucella* infection and were left to proceed until the time of analysis.

The transfection of small interfering RNA si GAPDH and siRNA control were performed on HeLa cells using the siPORT™ Amine transfection agent (Applied Biosystems Ambion) according to the manufacturer's instructions. The transfection of PKC ι, COP B, Rab 2 A, α-Enolase siRNAs and control siRNA-A were performed on HeLa cells using the siRNA Transfection Reagent (Santa Cruz Biotechnology) according to the manufacturer's instructions. 24 h later, transfected HeLa cells with the specific siRNA or siRNA-A control were infected with *B. abortus* as described before. To maintain the silencing of these specific proteins, HeLa cells were again transfected 2 h p.i.

Cellular extracts were prepared after lysis of HeLa cells transfected 72 h with either siGAPDH or siRNA control with 0.1% (vol/vol) Triton X-100 in PBS.

### Fractionation method

At 48 h p.i., 6 infected BHK-21 55 cm^2^ dishes were washed once with GMEM. 1 ml of GMEM was added and infected cells were recovered by scraping with a rubber policeman. Then, several steps of washes were done with PBS, PBS/1 mM EDTA, pH 7.4, homogenization buffer (3 mM imidazole/250 mM sucrose/0.5 mM EDTA/0.5 mM EGTA, pH 7.4). A centrifugation at 80 g for 5 min at 4°C was performed between each wash. Pellets were resuspended very gently in the homogenization buffer and cells were mechanically broken through 5 passages into a 22G needle. PNS were recovered after a centrifugation at 80 g for 10 min at 4°C. Then, a first step of purification was performed by loading the PNS on the top of a 50%–12% sucrose gradient. After centrifugation at 800 g for 45 min at 4°C, a cloudy layer containing BCVs appeared at the 50%–12% interface. This cloudy layer (called interface fraction) was carefully recovered and loaded at the bottom of a SW 60 centrifugation tube. Three layers of sucrose were sequentially added on top of the interface fraction: 30%, 20% and 5% sucrose respectively. The enriched BCV fraction was obtained after an ultracentrifugation at 35000 rpm for 1 h at 4°C and was localized in the pellet. The pellet was then resuspended in the homogenization buffer. Depending of experiments, a supplementary step was added between the two steps of sucrose gradient in order to eliminate mitochondria from the enriched BCV fraction. This step consisted of incubating the interface fraction with dynabeads (M-500 subcellular, Invitrogen) coated with a rabbit anti-VDAC 1 antibody (according to the manufacturer's instructions) overnight recovering the supernatant from dynabeads retained with a magnet (according to the manufacturer's instructions).

### Flow cytometry

BCV staining within PNS was adapted from what was previously described [40]. PNS was incubated with a rabbit anti-calnexin antibody and then incubated with a rabbit-PE antibody 30 min on ice. The preparation was fixed for 20 min in 3% final PFA and then diluted to 1% final PFA before analysis on a FACScalibur cytometer (Becton Dickinson). Data were analysed using FlowJo software (Tree Star).

### Preparation of 2D gel samples

250 µg of the enriched fraction of BCVs was treated for 30 min at room temperature with 0.1% Triton X-100. The BCV membranes were separated from bacteria by centrifugation at 10000 rpm for 5 min at 4°C. The corresponding supernatants enriched in BCV membrane proteins were precipitated with trichloroacetic acid (Sigma Aldrich) 10% final for 5 min on ice and then with trichloroacetic acid 5% final for 5 min on ice. Trichloroacetic acid was then removed by three washes with 90% acetone. Each step was followed by a centrifugation at 10000 rpm for 3 min at 4°C. The pellet was finally resuspended in 400 µl of destreack rehydration buffer containing 2% carrier ampholytes pH 3–10 (IPG Amersham Biosciences).

### 2-D gel electrophoresis

BCV membrane proteins were separated by isoelectric focusing (IEF). The IEF was performed using 18 cm gels with an immobilized linear pH gradient of 3–10 (Immobiline DryStrips, Amersham Biosciences) in IPGphor strip holders (Amersham Biosciences) on a MultiphorII machine (Amersham Biosciences). The IEF protocol was as follows: 300 V for 1 min; 500 V gradient for 30 min; 3500 V gradient for 1.5 h; 3500 V for 6 h. Temperature was set at 20°C. Prior to SDS PAGE, IPG strips were equilibrated during 20 min in an equilibration buffer (6 M urea, Tris, pH 8.8, 50 mM, 2% SDS, 65 mM DTT, 38.5% glycerol). The second dimension was performed using a Protean II xl Multicell separation unit (Biorad) and home-made 10% SDS PAGE gels. Temperature was set at 20°C. Gels, made of Tris-HCl, 0.1% SDS and 10% acrylamide were run at 20°C using the following running buffer (25 mM Tris, 192 mM glycine and 0.1% SDS) for the cathode part and 2×running buffer for the anode. Electrophoresis was conducted at 10 mA per gel overnight and stopped when the bromophenol blue front dye reached the bottom of the gel. Proteins were stained by a PlusOne Silver Staining Kit, Protein (GE Healthcare) according to the manufacturer's instructions (without glutaraldehyde to allow the mass spectrometry analysis).

### In-gel digestion and MALDI-TOF MS

Protein spots immediately excised from silver-stained gels were destained and subjected to in-gel digestion with trypsin (Sequencing grade modified porcine trypsine; Promega, Madison, WI, USA) according to a modified protocol from Shevchenko et al. [42]. Tryptic peptides were then extracted from the gel by successive treatment with 5% formic acid and 60% acetonitrile/5% formic acid. Extracts were pooled and dried in a Speedvac evaporator. Peptides resuspended in an α cyano-4-hydroxycinnamic acid matrix solution (prepared by diluting 6 times a saturated solution in 50% acetonitrile/0.3% trifluoroacetic acid), were then spotted on the metal target. Mass analyses were performed on a MALDI-TOF Bruker Ultraflex spectrometer (Bruker Daltonics, Wissembourg, France). Mass spectra were internally calibrated using autolytic peptides from trypsin.

### Database searching and data interpretation

The peptide mass lists were used to identify the protein using Mascot software available on site. Criteria used for protein identification are given by Mascot as a Probability Based Mowse Score. Ions score is −10*Log(P), where P is the probability that the observed match is a random event. Protein scores greater than X (X is a number between 60 and 74, from one search to the other) are significant (p<0.05).

### Western blotting

The protein concentration was determined with the BCA™ Protein Assay Kit (Pierce). Volumes corresponding to 60 µg of proteins from each main step of the fractionation were resuspended in 1×laemmeli-buffer and loaded on a 12% SDS polyacrylamide gel. Then proteins were transferred onto a PVDF membrane using a semi-dry transfer. The PVDF membrane was then blocked for 1 h with 4% milk/PBS/0.1% Tween 20 and incubated with different antibodies. The PVDF membrane was washed 3 times with PBS/0.1% Tween 20 before incubation with the secondary antibody and the detection. The detection was carried out using the ECL™ western blotting detection kit (Amersham).

### Fluorescence microscopy

To analyse the PNS and the sucrose step gradient enriched in BCVs, 10 µl of sample were put in 24-well plates containing 12-mm glass coverslips pre-treated with poly-L-lysine and incubated for 15 min at 37°C to allow the adherence onto glass coverslips. Then BCVs or infected cells were fixed with 3% paraformaldehyde, pH 7.4 at room temperature for 15 min, and then processed for immunofluorescence staining as described previously [5]. Specimens were observed on a Zeiss LSM 510 laser scanning confocal microscope for image acquisition. Images of 1024×1024 pixels were acquired and assembled using Adobe Photoshop CS2.

### Processing for electronic microscopy

*B. abortus*-infected BHK cells were fixed for 1 h at room temperature with 2.5% glutaraldehyde (Sigma, St Louis, MO, USA) in 0.1 M cacodylate buffer, pH 7.2, containing 0.1 M sucrose, 5 mM CaCl_2_ and 5 mM MgCl_2._ After two successive 15 min washes with the same buffer, cells were postfixed for 1 h at room temperature with 1% osmium tetroxide (Electron Microscopy Sciences, Hatfield, PA, USA) in the same buffer devoid of sucrose. The cells were scraped off the culture dishes with a rubber policeman and concentrated in 2% agarose in the same buffer. After 1 h incubation at room temperature with 1% uranyl acetate in veronal buffer, the samples were dehydrated in a graded series of acetone and embedded in Epon resin. Thin sections were stained with uranyl acetate and lead citrate. The PNS and enriched BCVs obtained from the BHK-infected cells were first prefixed for 20 min at room temperature with 5% glutaraldehyde in cacodylate buffer diluted at a 1∶1 volume ratio with the PNS or BCV fractions. These fractions were then processed as described above for BHK-infected cell.

## Supporting Information

Figure S1 Flow cytometry analysis of BCVs in the PNS: (A) BCV-GFP within the PNS is indicated by the GFP+ gate. (B) BCV-GFP subpopulation positive for the ER marker calnexin is indicated by the GFP+ ER+ gate. FL-1 indicates GFP fluorescence intensity and FL-2 indicates calnexin-PE fluorescence intensity.
(0.93 MB TIF)

Figure S2 The dominant negative of Rab 2 but not that of Rab 1 affects *B. abortus* replication. (A) Confocal micrographs of Hela cells infected with *B. abortus* Ds red and transfected or not with GFP, GFP Rab 1 or its dominant negative GFP Rab 1 S25N (Scale bars: 10 µm). (B) Confocal micrographs of Hela cells infected with *B. abortus* GFP and transfected with the dominant negative of Rab 2 (Rab 2 I 119) at 48 h p.i. Late endosomal compartments and the myc tag were immunostained with anti-LAMP-1 and anti-myc antibodies respectively (Scale bars: 10 µm).
(1.97 MB TIF)

Figure S3 Inhibition of expression of GAPDH prevents Rab2 to be recruited on BCVs. Purified BCVs from either control siRNA- or GAPDH siRNA-treated BHK cells were analysed by confocal microscopy for the presence of Rab2 (in red) immunostained with rabbit polyclonal anti-Rab2 antibody and LAMP-1 (in white) immunostained with mouse 4A1 monoclonal antibody (Scale bars: 5 µm).
(0.25 MB TIF)

Table S1 Proteic composition of isolated BCV: both eukaryotic and bacterial proteins are present on the BCV membrane. Each spot of the 2D gel electrophoresis of BCV membranes was analysed by mass spectrometry and identified by matching with the Mascot database software available on site.
(0.03 MB XLS)

## References

[1] Celli J, de Chastellier C, Franchini DM, Pizarro-Cerda J, Moreno E (2003). Brucella evades macrophage killing via VirB-dependent sustained interactions with the endoplasmic reticulum.. J Exp Med.

[2] Delrue RM, Martinez-Lorenzo M, Lestrate P, Danese I, Bielarz V (2001). Identification of Brucella spp. genes involved in intracellular trafficking.. Cell Microbiol.

[3] Detilleux PG, Deyoe BL, Cheville NF (1990). Penetration and intracellular growth of Brucella abortus in nonphagocytic cells in vitro.. Infect Immun.

[4] Liautard JP, Gross A, Dornand J, Kohler S (1996). Interactions between professional phagocytes and Brucella spp.. Microbiologia.

[5] Pizarro-Cerda J, Meresse S, Parton RG, van der Goot G, Sola-Landa A (1998). Brucella abortus transits through the autophagic pathway and replicates in the endoplasmic reticulum of nonprofessional phagocytes.. Infect Immun.

[6] Pizarro-Cerda J, Moreno E, Gorvel JP (2000). Invasion and intracellular trafficking of Brucella abortus in nonphagocytic cells.. Microbes Infect.

[7] Barlowe C (2002). COPII-dependent transport from the endoplasmic reticulum.. Curr Opin Cell Biol.

[8] Barlowe C, Orci L, Yeung T, Hosobuchi M, Hamamoto S (1994). COPII: a membrane coat formed by Sec proteins that drive vesicle budding from the endoplasmic reticulum.. Cell.

[9] Stephens DJ, Lin-Marq N, Pagano A, Pepperkok R, Paccaud JP (2000). COPI-coated ER-to-Golgi transport complexes segregate from COPII in close proximity to ER exit sites.. J Cell Sci.

[10] Celli J, Salcedo SP, Gorvel JP (2005). Brucella coopts the small GTPase Sar1 for intracellular replication.. Proc Natl Acad Sci U S A.

[11] Comerci DJ, Martinez-Lorenzo MJ, Sieira R, Gorvel JP, Ugalde RA (2001). Essential role of the VirB machinery in the maturation of the Brucella abortus-containing vacuole.. Cell Microbiol.

[12] Desjardins M, Huber LA, Parton RG, Griffiths G (1994). Biogenesis of phagolysosomes proceeds through a sequential series of interactions with the endocytic apparatus.. J Cell Biol.

[13] Garin J, Diez R, Kieffer S, Dermine JF, Duclos S (2001). The phagosome proteome: insight into phagosome functions.. J Cell Biol.

[14] Salcedo SP, Marchesini MI, Lelouard H, Fugier E, Jolly G (2008). Brucella control of dendritic cell maturation is dependent on the TIR-containing protein Btp1.. PLoS Pathog.

[15] Cloeckaert A, de Wergifosse P, Dubray G, Limet JN (1990). Identification of seven surface-exposed Brucella outer membrane proteins by use of monoclonal antibodies: immunogold labeling for electron microscopy and enzyme-linked immunosorbent assay.. Infect Immun.

[16] Chuang DM, Hough C, Senatorov VV (2005). Glyceraldehyde-3-phosphate dehydrogenase, apoptosis, and neurodegenerative diseases.. Annu Rev Pharmacol Toxicol.

[17] Sirover MA (1997). Role of the glycolytic protein, glyceraldehyde-3-phosphate dehydrogenase, in normal cell function and in cell pathology.. J Cell Biochem.

[18] Bryksin AV, Laktionov PP (2008). Role of glyceraldehyde-3-phosphate dehydrogenase in vesicular transport from golgi apparatus to endoplasmic reticulum.. Biochemistry (Mosc).

[19] Plutner H, Cox AD, Pind S, Khosravi-Far R, Bourne JR (1991). Rab1b regulates vesicular transport between the endoplasmic reticulum and successive Golgi compartments.. J Cell Biol.

[20] Robinson CG, Roy CR (2006). Attachment and fusion of endoplasmic reticulum with vacuoles containing Legionella pneumophila.. Cell Microbiol.

[21] Tisdale EJ, Bourne JR, Khosravi-Far R, Der CJ, Balch WE (1992). GTP-binding mutants of rab1 and rab2 are potent inhibitors of vesicular transport from the endoplasmic reticulum to the Golgi complex.. J Cell Biol.

[22] Derre I, Isberg RR (2004). Legionella pneumophila replication vacuole formation involves rapid recruitment of proteins of the early secretory system.. Infect Immun.

[23] Sirover MA (1999). New insights into an old protein: the functional diversity of mammalian glyceraldehyde-3-phosphate dehydrogenase.. Biochim Biophys Acta.

[24] Gruenberg J, van der Goot FG (2006). Mechanisms of pathogen entry through the endosomal compartments.. Nat Rev Mol Cell Biol.

[25] Schlumberger MC, Hardt WD (2006). Salmonella type III secretion effectors: pulling the host cell's strings.. Curr Opin Microbiol.

[26] Horwitz MA (1983). The Legionnaires' disease bacterium (Legionella pneumophila) inhibits phagosome-lysosome fusion in human monocytes.. J Exp Med.

[27] Shin S, Roy CR (2008). Host cell processes that influence the intracellular survival of Legionella pneumophila.. Cell Microbiol.

[28] Rogers LD, Foster LJ (2008). Contributions of proteomics to understanding phagosome maturation.. Cell Microbiol.

[29] Tisdale EJ (2001). Glyceraldehyde-3-phosphate dehydrogenase is required for vesicular transport in the early secretory pathway.. J Biol Chem.

[30] Tisdale EJ (2002). Glyceraldehyde-3-phosphate dehydrogenase is phosphorylated by protein kinase Ciota/lambda and plays a role in microtubule dynamics in the early secretory pathway.. J Biol Chem.

[31] Tisdale EJ (2003). Rab2 interacts directly with atypical protein kinase C (aPKC) iota/lambda and inhibits aPKCiota/lambda-dependent glyceraldehyde-3-phosphate dehydrogenase phosphorylation.. J Biol Chem.

[32] Tisdale EJ (2005). Rab2 purification and interaction with protein kinase C iota/lambda and glyceraldehyde-3-phosphate dehydrogenase.. Methods Enzymol.

[33] Tisdale EJ, Artalejo CR (2006). Src-dependent aprotein kinase C iota/lambda (aPKCiota/lambda) tyrosine phosphorylation is required for aPKCiota/lambda association with Rab2 and glyceraldehyde-3-phosphate dehydrogenase on pre-golgi intermediates.. J Biol Chem.

[34] Tisdale EJ, Artalejo CR (2007). A GAPDH mutant defective in Src-dependent tyrosine phosphorylation impedes Rab2-mediated events.. Traffic.

[35] Tisdale EJ, Kelly C, Artalejo CR (2004). Glyceraldehyde-3-phosphate dehydrogenase interacts with Rab2 and plays an essential role in endoplasmic reticulum to Golgi transport exclusive of its glycolytic activity.. J Biol Chem.

[36] Machner MP, Isberg RR (2006). Targeting of host Rab GTPase function by the intravacuolar pathogen Legionella pneumophila.. Dev Cell.

[37] Murata T, Delprato A, Ingmundson A, Toomre DK, Lambright DG (2006). The Legionella pneumophila effector protein DrrA is a Rab1 guanine nucleotide-exchange factor.. Nat Cell Biol.

[38] Ingmundson A, Delprato A, Lambright DG, Roy CR (2007). Legionella pneumophila proteins that regulate Rab1 membrane cycling.. Nature.

[39] Chen J, Reyes M, Clarke M, Shuman HA (2007). Host cell-dependent secretion and translocation of the LepA and LepB effectors of Legionella pneumophila.. Cell Microbiol.

[40] Meresse S, Andre P, Mishal Z, Barad M, Brun N (1997). Flow cytometric sorting and biochemical characterization of the late endosomal rab7-containing compartment.. Electrophoresis.

[41] de Chastellier C, Frehel C, Offredo C, Skamene E (1993). Implication of phagosome-lysosome fusion in restriction of Mycobacterium avium growth in bone marrow macrophages from genetically resistant mice.. Infect Immun.

[42] Shevchenko A, Wilm M, Vorm O, Mann M (1996). Mass spectrometric sequencing of proteins silver-stained polyacrylamide gels.. Anal Chem.

[43] Cloeckaert A, Zygmunt MS, Bezard G, Dubray G (1996). Purification and antigenic analysis of the major 25-kilodalton outer membrane protein of Brucella abortus.. Res Microbiol.

