# Supplementary figures and images for: The Glyceraldehyde-3-Phosphate Dehydrogenase and the Small GTPase Rab 2 Are Crucial for Brucella Replication
Source: PLoS Pathog. 2009 Jun 26;5(6):e1000487. doi: 10.1371/journal.ppat.1000487 (PMC2695806; doi:10.1371/journal.ppat.1000487)

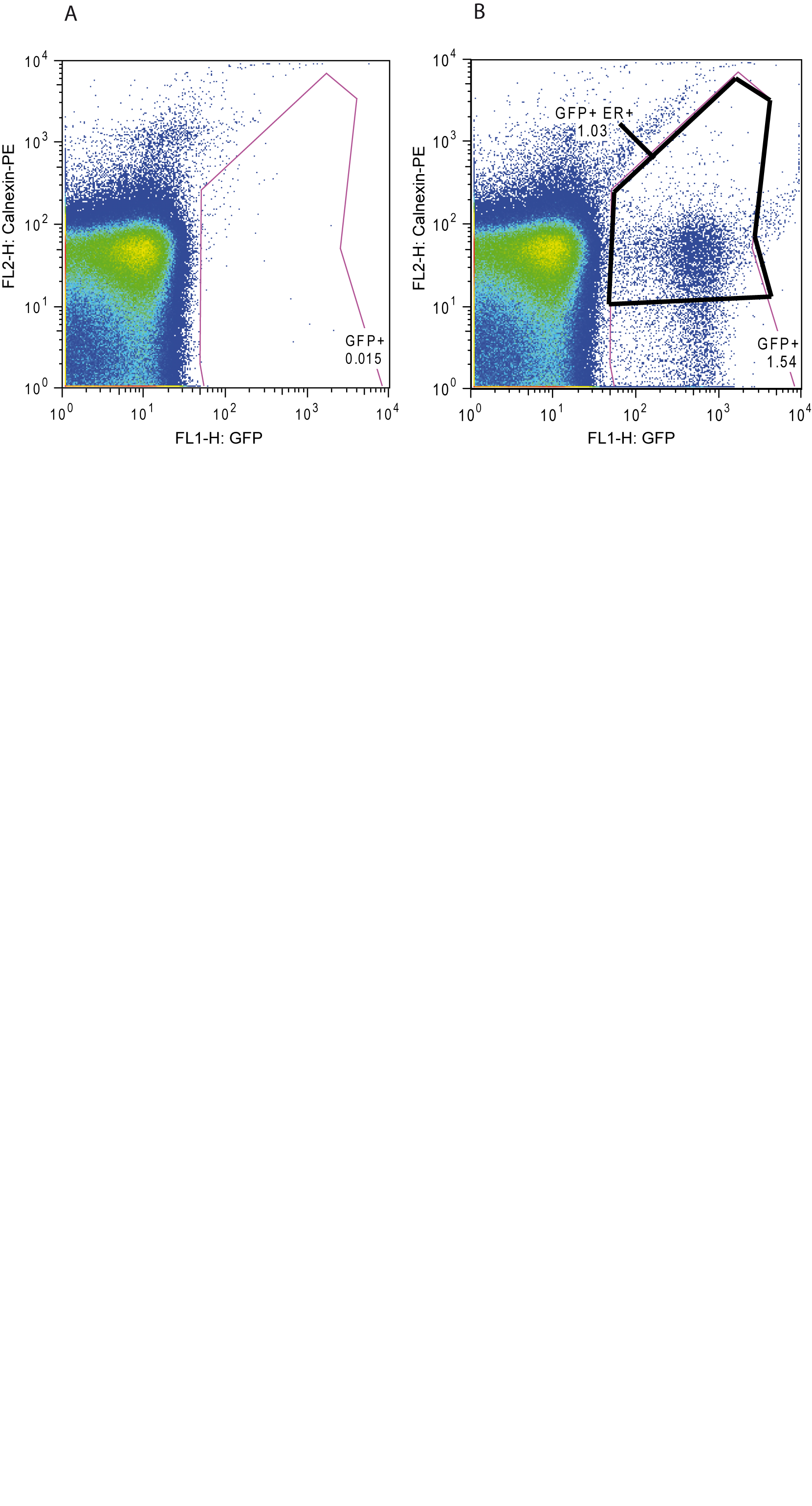

Supplement: Figure S1 — Flow cytometry analysis of BCVs in the PNS: (A) BCV-GFP within the PNS is indicated by the GFP+ gate. (B) BCV-GFP subpopulation positive for the ER marker calnexin is indicated by the GFP+ ER+ gate. FL-1 indicates GFP fluorescence intensity and FL-2 indicates calnexin-PE fluorescence intensity. (0.93 MB TIF) [file ppat.1000487.s001.tif]

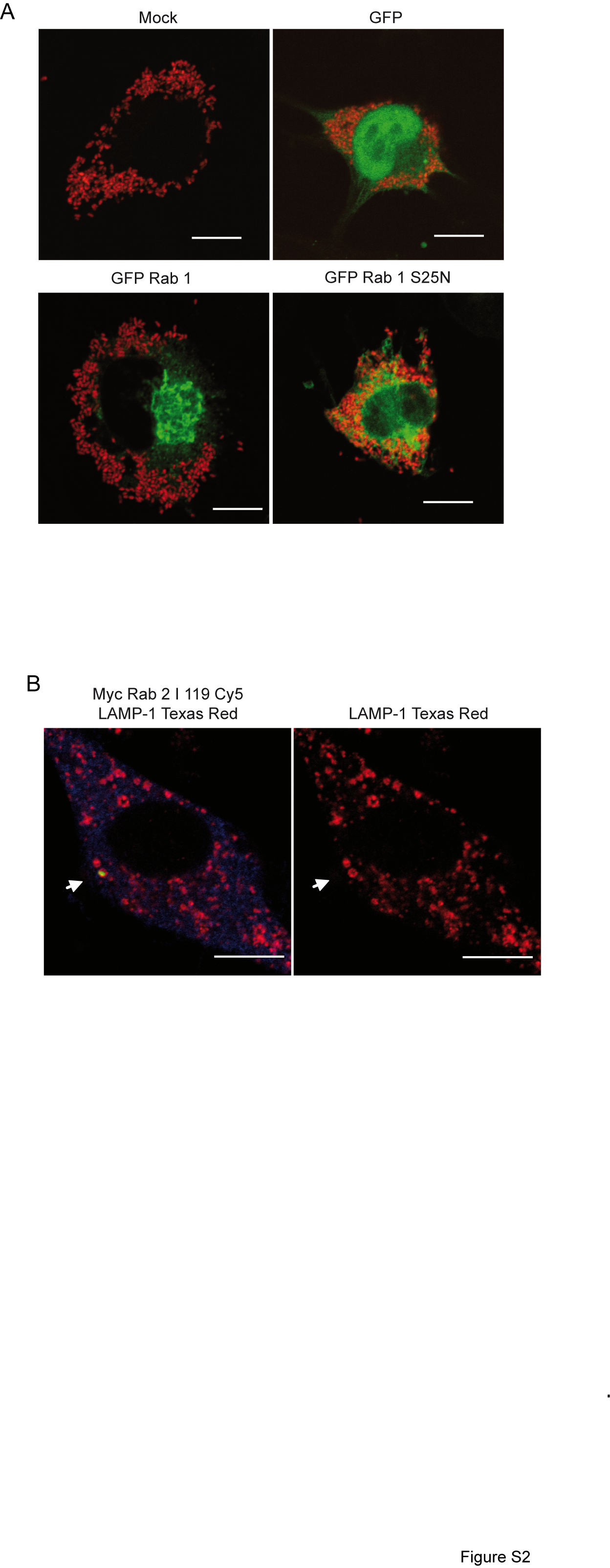

Supplement: Figure S2 — The dominant negative of Rab 2 but not that of Rab 1 affects B. abortus replication. (A) Confocal micrographs of Hela cells infected with B. abortus Ds red and transfected or not with GFP, GFP Rab 1 or its dominant negative GFP Rab 1 S25N (Scale bars: 10 µm). (B) Confocal micrographs of Hela cells infected with B. abortus GFP and transfected with the dominant negative of Rab 2 (Rab 2 I 119) at 48 h p.i. Late endosomal compartments and the myc tag were immunostained with anti-LAMP-1 and anti-myc antibodies respectively (Scale bars: 10 µm). (1.97 MB TIF) [file ppat.1000487.s002.tif]

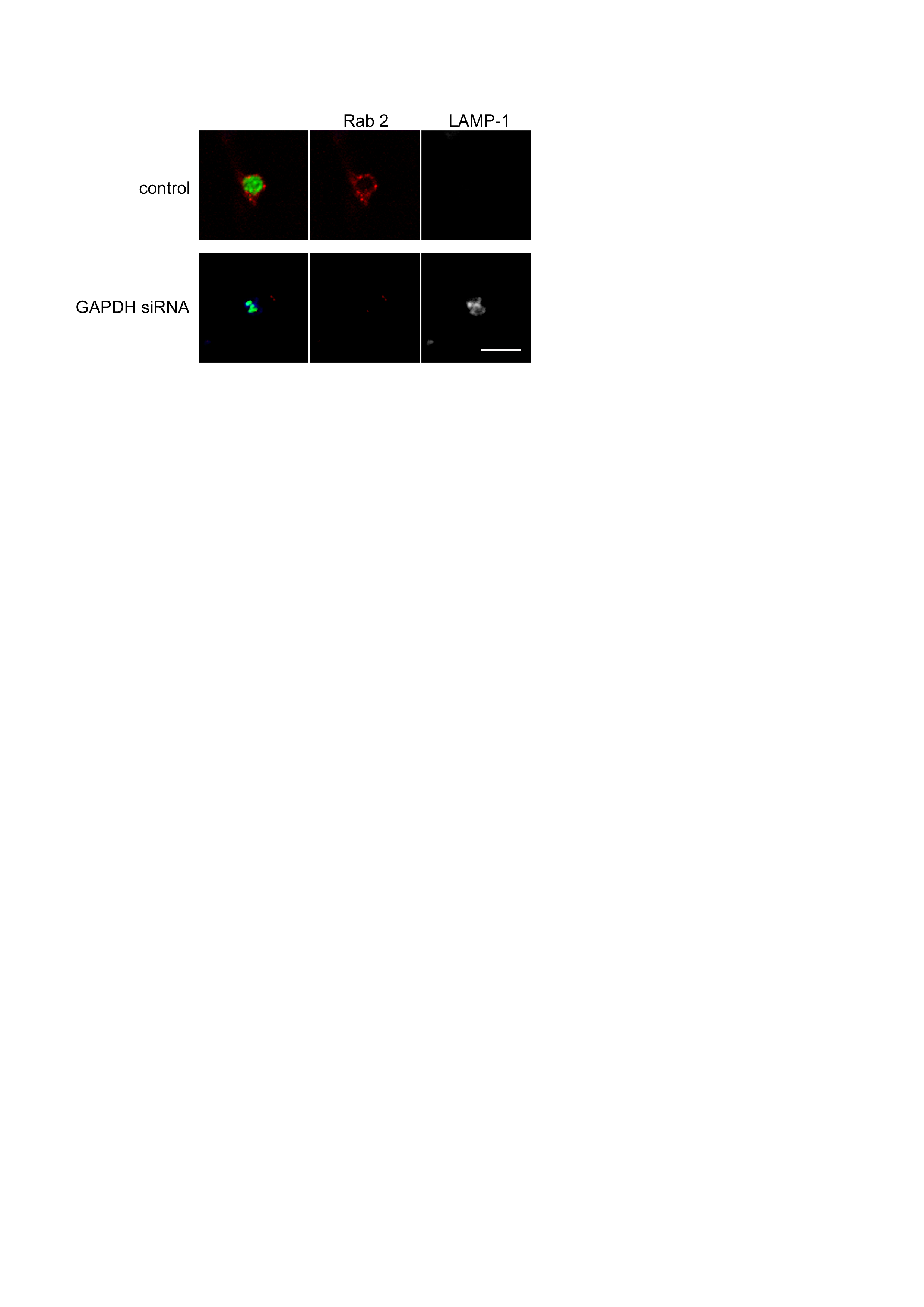

Supplement: Figure S3 — Inhibition of expression of GAPDH prevents Rab2 to be recruited on BCVs. Purified BCVs from either control siRNA- or GAPDH siRNA-treated BHK cells were analysed by confocal microscopy for the presence of Rab2 (in red) immunostained with rabbit polyclonal anti-Rab2 antibody and LAMP-1 (in white) immunostained with mouse 4A1 monoclonal antibody (Scale bars: 5 µm). (0.25 MB TIF) [file ppat.1000487.s003.tif]
